# Validity and reliability of the Japanese version of the Patient Satisfaction Questionnaire (PSQ-J) for evaluating oncologist consultations

**DOI:** 10.1016/j.pecinn.2023.100166

**Published:** 2023-05-18

**Authors:** Yuiko Kamo, Maiko Fujimori, Mariko Asai, Takayuki Oishi, Masanori Mori, Mitsunori Miyashita, Tatsuya Morita, Yosuke Uchitomi

**Affiliations:** a Department Division of Supportive Care, Survivorship and Translational Research, National Cancer Center Institute for Cancer Control, Tokyo, Japan; b Department of Medical Psychology, Nippon Medical School, Tokyo, Japan; c Department of Clinical Oncology, Institute of Development, Aging and Cancer, Tohoku University, Miyagi, Japan; d Department of Palliative Medicine, Seirei Hamamatsu General Hospital, Shizuoka, Japan; e Department of Palliative Nursing, Tohoku University Graduate School of Medicine, Miyagi, Japan; f National Cancer Center Institute for Cancer Control, Tokyo, Japan

**Keywords:** Satisfaction, Physician-patient relations, Communication, Psychometrics, Oncology

## Abstract

**Objective:**

To develop the Japanese version of the Patient Satisfaction Questionnaire (PSQ-J) and examine its validity and reliability.

**Methods:**

A cross-sectional, web-based survey was conducted among cancer patients in Japan. The PSQ-J was developed following the forward-backward translation method, using a numerical rating scale. Data on patient characteristics and psychometric scales, like the PSQ-J, willingness to recommend an oncologist to others, trust in the healthcare system, uncertainty, and the Physician Compassion Questionnaire were collected. Validity was examined using exploratory and confirmatory factor analyses and by calculating the correlations between the total PSQ-J score and the criterion variables. Reliability was confirmed by Cronbach's alpha and test-retest score correlations at a two-week interval.

**Results:**

The first and second surveys were conducted on 309 and 107 patients, respectively. One-dimensionality and model fit were verified using factor analyses. The PSQ-J was significantly associated with other comparable scales. Cronbach's alpha was 0.962; the correlation between the PSQ-J test-retest scores was 0.835 (*p* < .001).

**Conclusion:**

The current study indicates that the PSQ-J can be valid and reliable for assessing satisfaction with oncologist consultation.

**Innovation:**

The PSQ-J enables the effective assessment of patient satisfaction with oncologist consultations, leading to better practice reflecting the patient's viewpoint.

## Highlights

• To evaluate patients' satisfaction with oncologists' communication is important.
• The Japanese version of the Patient Satisfaction Questionnaire was developed.
• The questionnaire's validity and reliability were confirmed in Japanese patients.

## 1 Introduction

Patient satisfaction is based on the evaluation of the process of medical provision and quality of care. It embodies patients' perceptual components, such as their needs, expectations for the health system, and healthcare experiences [1,2]. In particular, when facing a severe disease such as cancer, patient satisfaction with medical care can affect health outcomes. For instance, it has been reported that high patient satisfaction with medical care improved the effectiveness of treatment and patients' quality of life or relieved their distress [1,3,4]. Thus, assessing patient satisfaction is crucial to achieving appropriate medical provisions and patient outcomes.

There are various patient satisfaction indicators of patient satisfaction, of which the Patients-Satisfaction Questionnaire (PSQ) focuses on how satisfied cancer patients are with their oncologists' consultations. The questionnaire asked about patient satisfaction using five items: 1) the way the doctor addresses your needs; 2) your active involvement in the interaction with the doctor; 3) information received from the doctor; 4) emotional support from the doctor; and 5) the interaction in general. These items were selected based on previous surveys and measured using a visual analog scale (VAS: 0 to 100) [4,5].

Several studies have adopted the PSQ; one of these studies examined the difference in subjective and objective perceptions of patient satisfaction with doctor consultation, assessed the validity of other psychological measurements, and evaluated the doctor's communication quality [[5], [6], [7]]. High internal reliability (Cronbach's alpha) of the PSQ (0.900) was reported in the literature [5]. The PSQ has also been used in Japan; however, the authors could not find any study that meticulously examined the validity and reliability of the Japanese version of the PSQ (PSQ-J) [8]. Specifically, the use of the numerical rating scale (NRS: 0 to 10) to assess the PSQ-J was not found, although the NRS is thought to be more commonly used in Japan [9]. Therefore, this study aimed to examine the reliability and validity of the PSQ-J on the NRS in Japanese patients with cancer. Considering its validity and reliability in this study, valuable insights into the PSQ-J based on the NRS can be obtained for future use. Moreover, the interpretation of its scores will help evaluate oncologists' consultations.

## 2 Material and methods

### 2.1 Participants and procedure

First, an expert and a bilingual developed the PSQ-J using the forward–backward translation method. Second, a cross-sectional web-based survey including the PSQ-J was conducted. The inclusion criteria included patients with a cancer diagnosis, who visited a doctor for cancer treatment, and who were registered with an outsourced survey company. The target number of participants was 300 for the test and 100 for the retest. The validation sample size was estimated to be 2–20 individuals per item, with an absolute minimum of 100–250 [10]. Therefore, we determined that the required sample size was at least 250. The web-based survey was closed when the target number was reached. The authors first obtained data via a web-based questionnaire and requested participants to answer the same psychological instruments for the test-retest method after two weeks. The validity and reliability were verified following previous studies [6,11]. This study was permitted by the original developer of the PSQ and approved by the Research Ethics Committee of Seirei Mikatahara General Hospital [4]. The participants were informed about the study's purpose, the voluntary nature of participation, and privacy protection policies. Additionally, e-consent was obtained from them on the website.

### 2.2 Variables

#### 2.2.1 Instruments

##### 2.2.1.1 Patient satisfaction with oncologist consultation

The five-item PSQ-J measured patient satisfaction with oncologist consultation [4,5]. The participants were asked the following five questions: (1) How well did the doctor address your needs? (2) How actively were you involved in talking and participating in the interaction? (3) How satisfied are you with the adequacy of the information received from your doctor? (4) How satisfied are you with the emotional support you receive from your doctor? (5) Overall, how satisfied are you with the interaction? Each item was rated on an 11-point Likert scale (not at all satisfied = 0 to a great deal of satisfaction = 10). A higher score indicated a higher level of satisfaction, and the total score of the five items was calculated to assess overall patient satisfaction.

##### 2.2.1.2 Willingness to recommend the oncologist to others

This was assessed using one item: Would you recommend the doctor to a patient you know? In the questionnaire, the answers were obtained on a five-point Likert scale (strongly disagree = 1 to strongly agree = 5).

##### 2.2.1.3 Trust in the healthcare system

This was assessed with one item, rated on a five-point Likert scale (very little trust = 1 to a great deal of trust = 5): How much do you trust the current Japanese healthcare system?

##### 2.2.1.4 Uncertainty

Uncertainty was assessed using one item: How uncertain (lack of visibility) you felt about your future due to the physician's communication? It was rated on an 11-point Likert scale (a great deal of certainty = 0 to a great deal of uncertainty = 10). Lower scores indicated a positive prospect [9,12].

##### 2.2.1.5 Physician Compassion Questionnaire

This scale measured the patient's perception of the physician's empathic attitude, which consists of five items: ‘warm’ – ‘cold’, ‘pleasant’ – ‘unpleasant’, ‘compassionate’ – ‘distant’, ‘sensitive’ – ‘insensitive’, and ‘caring’ – ‘uncaring’. Each item was rated on a scale of 0–10, and physicians' empathy was indicated by a score of 0–50. These scores are interpreted inversely, with lower scores indicating higher physician empathy [[13], [14], [15]].

#### 2.2.2 Patient characteristics

The survey collected data on patient characteristics such as age, gender, marital status, parental status, job status, household income, length of time since their cancer diagnosis, quality of life (Functional Assessment of Cancer Therapy-General: FACT-G) [16], the expertise of the oncologist, and the number of oncologist consultations.

### 2.3 Statistical analysis

The validity and reliability of the PSQ-J were determined on the NRS. First, descriptive statistical analyses were performed. Then, whether there were any extreme biases, ceiling effects, or floor effects for each item was confirmed. Second, the structural and concurrent validity was confirmed. Structural validity was determined by performing exploratory factor analysis with orthogonal rotation (varimax) to allow for factors to load on more than one factor; thus, not presuming one-dimensionality. An eigenvalue cutoff of >1.0 was used to indicate different factors. A confirmatory factor analysis was also performed to evaluate the goodness-of-fit of the PSQ original factor model using chi-square (*χ*^*2*^), where a small, non-significant statistic indicates a good model-data fit. However, non-significant chi-square values are seldom obtained for a large sample. Thus, other fit indices were also evaluated: (1) the goodness-of-fit index (GFI); (2) the adjusted goodness-of-fit index (AGFI); (3) the comparative fit index (CFI); (4) the root mean square error of approximation (RMSEA); and (5) the standardised root mean residual (SRMR). The values of these indices range from 0 to 1. The acceptable fit citation for the GFI, AGFI, and CFI was adequate at >0.900. For the RMSEA and SRMR, values <0.080 indicated a good fit. Furthermore, to establish concurrent validity, correlation coefficients were calculated and secured on the assumption that the items were positively correlated with the related variables. Correlation coefficients were evaluated using the following scales: willingness to recommend the oncologist to others (1–5 points), trust in the healthcare system (1–5 points), uncertainty (0–11 points), Physician Compassion Questionnaire (0–50 points), and the number of previous consultations. It was expected that high satisfaction would be strongly associated with low uncertainty and the Physician Compassion Questionnaire and high willingness to recommend the oncologist to others would be moderately associated with high trust in the healthcare system and a more significant number of previous consultations [6,11,17,18]. Finally, reliability was assessed by examining internal consistency and reproducibility using the test-retest method. Cronbach's alpha and correlations between the total scores of the first and second (two weeks later) PSQ-J were calculated. Spearman's ordinate correlation coefficient was used to examine the associations. Data were analysed with SPSS version 27.0 and Amos version 26.0 (IBM). A significant difference was defined at *p* < .050.

## 3 Results

### 3.1 Participants

A total of 633 eligible registrants received e-mail requests for the first survey; the survey was closed when 309 (49%) responses were received. Two weeks later, 157 who participated in the first survey were invited to the second one, with 109 (66%) responding to the second survey. Table 1 shows the descriptive statistics of participant characteristics. The median age was 58 years old, and there were 173 males and 136 females (56.0% vs. 44.0%) in the sample. Most participants (75.1%) got their cancer diagnosis more than two years ago. The mean FACT-G scores were as follows: physical well-being 22.9, social/family well-being 13.4, emotional well-being 16.4, and functional well-being 17.6. The most common specialty of their oncologists was breast medicine and surgery (25.2%), followed by pulmonary or gastrointestinal surgery (23.3%) and urology (17.5%); the number of oncologist consultations was 16 or more, and most participants had seen their doctors for more than two years.

**Table 1:** Characteristics of the participants (*n* = 309).

Variable	*N*	%	Mean (SD)	Median	Range
Age (*n* = 309)			56.8 (8.3)	58	40–69
Gender					
Male	173	56.0			
Female	136	44.0			
Marital status (*n* = 309)					
Married	232	75.1			
Not married	77	24.9			
Parental Status (*n* = 309)					
No child	93	30.1			
Have child(ren)	216	69.9			
House income (*n* = 270)					
Under 2 million yen per year	20	6.7			
2 million to 10 million yen per year	214	71.6			
>10 million yen per year	36	12.0			
Job status (*n* = 309)					
Have occupation	149	48.2			
Have part-time occupation	33	10.7			
No occupation	127	41.1			
QOL (FACT-G)					
Physical [0–28] (*n* = 309)			22.9 (5.0)		1–28
Social/family [0–28] (*n* = 292)			13.4 (6.0)		1–27
Emotion [0–24] (*n* = 309)			16.4 (4.8)		1–24
Function [0–28] (*n* = 309)			17.6 (6.1)		1–28
Length of time since the cancer diagnosis (*n* = 309)					
One month to less than six months	5	1.6			
Six months to less than two years	72	23.3			
More than two years	232	75.1			
Expertise of the oncologist (*n* = 309)					
Breast medicine and surgery	78	25.3			
Pulmonary or gastrointestinal surgery	72	23.3			
Urology	54	17.5			
Haematology	18	5.8			
Gastroenterology	26	8.4			
Gynaecology	15	4.9			
Respiratory medicine	10	3.2			
Otolaryngology or otorhinolaryngology	8	2.6			
Oncology chemotherapy	6	1.9			
Radiotherapy	2	0.6			
Others or I don't know	20	6.5			
Number of oncologist consultations (*n* = 306)					
<3 times	4	1.3			
3–5 times	9	2.9			
6–10 times	32	10.4			
11–15 times	35	11.3			
16≤	226	73.1			
Length of consultation by the doctor for cancer treatment (*n* = 308)					
Less than one month	4	1.3			
One month to less than six months	12	3.9			
Six months to less than two years	85	27.5			
More than two years	207	67.0			

### 3.2 PSQ-J scores

Table 2 shows the descriptive statistics for each item on the PSQ-J. The scores of patient satisfaction with the way the doctor addressed patient needs and information received from the doctor were the highest, whereas emotional support from the doctor was the lowest in the test and retest PSQ-J. All item score distributions were between −0.78 and −0.23 and were not found to be highly skewed.

**Table 2:** Overview of all the items of the PSQ: mean, median, range, skewness, and factor loading.

	Mean (SD)		Median		Range		Skewness		Factor loading
	Test	retest	Test	retest	Test	retest	test	retest	
PSQ-J each item score									
1) the way the doctor addresses your need	7.21(1.85)	7.43(1.84)	8	8	2–10	2–10	−0.52	−0.54	0.93
2) your active involvement in the interaction with the doctor	7.14(1.96)	7.44(1.93)	7	8	0–10	3–10	−0.53	−0.39	0.84
3) information received from the doctor	7.21(1.86)	7.31(1.85)	7	8	1–10	2–10	−0.50	−0.55	0.92
4) emotional support from the doctor	6.72(2.13)	6.76(2.09)	7	7	0–10	2–10	−0.61	−0.23	0.93
5) the interaction in general	7.04(2.11)	7.21(2.03)	7	8	0–10	2–10	−0.78	−0.64	0.97
Total PSQ-J score	35.32(9.26)	36.15(9.19)	37	38	5–50	12–50	−0.50	−0.46	

Note. PSQ = Patient Satisfactory Questionnaire, SD = Standard Deviation, NRS: not at all satisfied = 0 to extremely satisfied = 10.

Factor loading: using varimax.

### 3.3 Validity

Table 2 shows the results of the exploratory factor analysis. The exploratory factor analysis of the PSQ-J indicated a one-factor solution. Table 3 shows the results of the confirmatory factor analysis. The overall goodness-of-fit chi-square statistic was 30.544. The GFI (0.963) and CFI (0.987) were >0.900, and the AGFI (0.888) was fairly close to 0.900. The RMSEA (0.129) was >0.080, whereas the SRMR (0.014) was <0.080, indicating that the GFI, CFI, AGFI, and SRMR criterion values have a reasonable fit. Overall, the fit indices were regarded to have an adequate fit of data to the original PSQ questionnaire model.

**Table 3:** Goodness of fit of the PSQ questionnaire factor model.

Index	Value
*χ*^2^ (df), *p*-value	30.544(5), *p* < .001
GFI	0.963
AGFI	0.888
CFI	0.987
RMSEA (90% Cls)	0.129 (0.087**–0**.174)
SRMR	0.014

GFI: goodness-of-fit index; AGFI: adjusted goodness-of-fit index; CFI: comparative fit index; RMSEA: root mean square error of approximation; SRMR: standardised root mean residual; Cls: confidence intervals.

Table 4 indicates the results of concurrent validity. Spearman's ordinate correlation coefficients between the PSQ-J and the criterion variables were calculated. The willingness to recommend the oncologist to others (*r* = 0.561, *p* < .001) and trust in the healthcare system (*r* = 0.380, *p* < .001) scales were positively associated with the PSQ-J, whereas uncertainty (*r* = −0.620, p < .001) and the Physician Compassion questionnaire (*r* = −0.713, p < .001) scales demonstrated negative correlations. The number of previous consultations with the oncologist was not significantly associated with the PSQ-J (*r* = 0.001 *p* < .982). These associations were almost consistent with the predictions based on the meaning of each instrument.

**Table 4:** Correlations between PSQ scores and willingness to recommend the oncologist to others, trust in the healthcare system, uncertainty, physician compassion questionnaire, and number of previous consultations with the oncologist.

	Willingness to recommend the oncologist to others	Trust in the healthcare system	Uncertainty	Physician Compassion Questionnaire	Number of previous consultations with the oncologist^**†**^
PSQ-J total score	0.560**	0.380**	−0.620**	−0.713**	−0.001

Note. ^**†**^ Five-point Likert scale: 1: 1–2, 2: 3–5, 3: 6–10, 4: 11–15, 5: ≥16, **p < .010.

### 3.4 Reliability

Table 5 shows the results of Cronbach's alpha and correlations between the PSQ-J test-retest total scores. Cronbach's alpha was 0.962 in this study. Additionally, the correlation related to test-retest reliability was 0.835 (*p* < .001) by comparing the total scores of the PSQ-J in the first and second surveys.

**Table 5:** Cronbach's alpha and correlations between PSQ-J test-retest total scores.

	Cronbach's alpha	Correlation between test-retest PSQ-J total scores
PSQ-J total score	0.962	0.835**

Note. PSQ = Patient-Satisfaction Questionnaire, SD = Standard Deviation, **p < .010.

## 4 Discussion and conclusions

### 4.1 Discussion

The Japanese version of the PSQ was developed and standardised in this study. No ceiling or floor effects were observed in the distribution, and the results indicated high validity and reliability. The study results showed that the item score of emotional support was lower than that in the Netherlands (6.7/10 points vs. 80/100 points, respectively) [5]. Additionally, the total score of the PSQ-J was lower overall than those in studies abroad (35.3/50 points vs. 81/100 points and 82.7/100 points, respectively) [5,11]. No reason for the comparably lower PSQ-J scores in Japan than other countries could be specified because the timing of responses, which can affect the scores due to recall bias, was not clear in this study.

Exploratory factor and conformity analyses indicated a one-factor structure. Therefore, this model was collectively adequate. As expected, the concurrent validity results collectively matched for all criterion variables except for the number of previous oncology consultations. This result is reasonable because the original study of the PSQ reported that the PSQ was not associated with the number of prior consultations [5]. Thus, the PSQ-J was adequate for both construct and concurrent validity.

Concerning reliability, the internal consistency of the PSQ-J (Cronbach's alpha = 0.962) was found to be excellent. This result is consistent with a previous study, which reported a Cronbach's alpha of the PSQ of 0.900 [5]. Additionally, test-retest reliability was determined by calculating the correlations of the PSQ-J in the first and second surveys. The correlation between the test-retest PSQ-J total scores was 0.835 (*p* < .001). This indicates acceptable reproducibility [19]. Consequently, the results demonstrated high reliability of the PSQ-J on the NRS.

This study contributes to effectively assessing oncologist consultations. Nonetheless, it had several limitations. First, recall biases were likely to occur in the responses, as the authors did not know the time that had elapsed since the participant visited their oncologist. In previous studies, the questionnaires, including the PSQ, were administered to participants just a few days after the consultation. Further investigation is needed while considering the timing of the PSQ-J question. Second, there may have been a selection bias. The population in the adolescent and young adult groups was not included, and the participants were likely to see their oncologists many times. Finally, we did not change the order of the PSQ-J items for the retest. Although we retested two weeks later, the testing effect from participants could occur and lead to an influence on responses. Despite these limitations, this study is the first to develop the PSQ-J and analyse its validity and reliability. Using an NRS, we have provided evidence that the PSQ-J is valid and reliable for measuring cancer patients' satisfaction with doctor consultations in Japan.

### 4.2 Innovation

The PSQ-J validated in this study contributes to effectively assessing oncologist consultations from the patients' perspective. Moreover, the NRS facilitated the use of various surveys, including web-based surveys. These contributions will facilitate assessing the quality of general practices and will develop and evaluate interventions for improving oncologists' consultation by reflecting the view of Japanese cancer patients.

### 4.3 Conclusion

The current study confirms that the PSQ-J is valid and reliable for assessing satisfaction with oncologist consultations in Japan. However, further research is needed to examine the validity and reliability of the PSQ-J with other groups, including adolescents and young adults.

## CRediT authorship contribution statement

Yuiko Kamo: Formal analysis, Interpretation of data, Writing – original draft. Maiko Fujimori: Formal analysis, Interpretation of data, Writing – review & editing, Supervision. Mariko Asai: Formal analysis, Interpretation of data, Writing – review & editing, Supervision. Takayuki Oishi: Conceptualization, Methodology, Writing – review & editing, Masanori Mori: Conceptualization, Methodology, Writing – review & editing, Mitsunori Miyashita: Conceptualization, Methodology, Writing – review & editing, Tatsuya Morita: Conceptualization, Methodology, Writing – review & editing, Yosuke Uchitomi: Writing – review & editing, Supervision. All authors have read and approved the final manuscript.

## Funding

This study was supported by 10.13039/501100001691JSPS KAKENHI (grant number JP19H03878).

## Declaration of Competing Interest

None.

## Data Availability

The datasets used and/or analysed during the current study are available from the corresponding author upon reasonable request.
